# Theoretical Investigation of Auger and Electron–Surface Optical Phonon Processes near the K-Points in Monolayer PtSe_2_ and PtS_2_ on Polar Dielectric Substrates

**DOI:** 10.3390/ma19071280

**Published:** 2026-03-24

**Authors:** Mounira Mahdouani, Amine Oudir, Spiros Gardelis, Ramzi Bourguiga

**Affiliations:** 1 Laboratoire de Physique des Matériaux Structure et Propriétés (LR01ES15), Groupe Physique des Composants et Dispositifs Nanométriques, Faculté des Sciences de Bizerte, Université de Carthage, Jarzouna-Bizerte 7021, Tunisia; mounira.mahdouani@ipeib.ucar.tn (M.M.); amine.oudir@issatm.ucar.tn (A.O.); ramzi.bourguiga@fsb.ucar.tn (R.B.); 2 Condensed Matter Physics Section, Physics Department, National and Kapodistrian University of Athens, Panepistimiopolis, Zografos, 15784 Athens, Greece

**Keywords:** Auger recombination, Pt-based van der Waals heterostructures, surface optical phonons, polaronic oscillator strength, polaronic scattering rate

## Abstract

In this work, we present a theoretical investigation of electron–surface optical phonon (SOP) interactions and Auger recombination processes in monolayer PtSe_2_ and PtS_2_ supported on polar dielectric substrates such as SiO_2_ and hBN. The analysis is based on a low-energy effective Hamiltonian describing the electronic structure near the K and K′ valleys of the Brillouin zone, combined with the Fröhlich interaction model to account for the coupling between charge carriers and substrate-induced optical phonons. The comparison between Auger recombination and SOP scattering is performed at a representative carrier density of ${n=10}^{12}\;{cm}^{-2}$ within the investigated temperature range. We analyze the formation of polaronic states arising from the hybridization between electronic excitations and SOPs and evaluate the associated Rabi splitting energies and oscillator strengths. The temperature dependence of the SOP-induced scattering rates and the influence of the monolayer–substrate separation on carrier–phonon interactions are also examined. Our results show that electron–phonon coupling strongly depends on the dielectric properties of the supporting substrate, with larger anticrossing gaps predicted for hBN-supported structures compared with SiO_2_-supported systems. Auger recombination constitutes the dominant carrier relaxation channel within the investigated temperature range, whereas SOP scattering becomes increasingly significant at elevated temperatures, where both mechanisms approach a comparable inelastic phonon-limited regime. These findings highlight the role of dielectric engineering in controlling carrier relaxation dynamics in Pt-based TMDC heterostructures.

## 1. Introduction

Group-X transition metal dichalcogenides (TMDCs), with the chemical formula MX_2_ (M = Pt or Pd; X = S, Se, or Te), have attracted substantial interest owing to their tunable bandgaps, which span from the visible region in the monolayer limit to the mid-infrared range in multilayer configurations [1]. These compounds also possess high electron mobility [2] and remarkable chemical stability under both ambient and aqueous environments [3,4], positioning them as promising candidates for diverse optoelectronic technologies.

Carrier mobility in group-X TMDCs such as PtSe_2_ and PtS_2_ typically ranges from tens to several hundred cm^2^ V^−1^ s^−1^ depending on layer thickness, crystal quality, and substrate environment [2]. These mobility values are comparable to those observed in many layered semiconductor systems and are sufficiently high for applications in field-effect transistors, photodetectors, and other optoelectronic devices.

Their distinctive characteristics have enabled significant progress in applications such as broadband photodetectors [5], light-emitting devices [6], field-effect transistors [7], label-free biosensors [4,8], holographic platforms [9], and nanoscale optical elements, including ultrathin lenses [10]. Consequently, a comprehensive understanding of key optical properties—including optical absorption, exciton dynamics, photoluminescence efficiency, and carrier relaxation mechanisms—is essential for the development and optimization of next-generation optoelectronic device architectures.

Within this family, PtSe_2_ and PtS_2_ are particularly notable for their versatile electronic and optical responses due to several intrinsic physical properties. These include a strong layer-dependent electronic structure, tunable band gaps, and significant spin–orbit coupling effects. In addition, dielectric screening and interfacial interactions in supported heterostructures can strongly modify their optical and electronic behavior. As a result, these materials have attracted considerable attention for applications in photodetectors, sensors, and optoelectronic devices [11,12].

Group-X TMDCs, including PtSe_2_ and PtS_2_, typically exhibit band gaps in the range of approximately 0.2–1.2 eV, depending on the number of layers and external conditions such as strain or dielectric environment. This relatively narrow band-gap range makes these materials particularly attractive for infrared photodetectors and optoelectronic applications.

Narrow band-gap semiconductors such as Bi_2_Te_3_-based materials have attracted considerable attention due to their remarkable thermoelectric and optoelectronic properties. Recent studies have demonstrated that Bi_2_Te_3_-based systems exhibit strong carrier transport and optical responses associated with their narrow band gaps and topological electronic structure [13]. In comparison, TMDCs such as PtSe_2_ and PtS_2_ offer complementary advantages arising from their two-dimensional layered structure and tunable electronic properties. Their band gaps can be modified through layer thickness, strain, and dielectric environment, making them promising candidates for infrared photodetection and optoelectronic applications.

PtSe_2_ exhibits a strongly thickness-dependent electronic structure, evolving from a semiconducting phase in the monolayer to a semimetallic phase in bulk. This tunability allows precise control over its conductivity and bandgap, supporting its integration into nanoelectronic systems. Its high carrier mobility combined with pronounced spin–orbit coupling further highlight its potential in logic devices, photodetectors, and spintronic applications [14]. Conversely, PtS_2_ maintains semiconducting behavior even in multilayer form, characterized by an indirect bandgap and pronounced photoresponsivity under visible light illumination [15,16]. Both compounds exhibit excellent environmental stability, a prerequisite for practical device operation. Optically, PtSe_2_ and PtS_2_ are marked by strong light–matter interactions, large absorption coefficients across the visible to near-infrared spectrum, and ultrafast photoresponse, making them highly suitable for broadband photodetection, optical modulation, and flexible sensing platforms [15,16]. In addition, their integration into layered heterostructures provides further opportunities to tailor their optical response through interlayer coupling and dielectric engineering.

The influence of dielectric substrates, particularly polar insulators such as SiO_2_ and hBN, plays a central role in the design of van der Waals heterostructures incorporating PtSe_2_ and PtS_2_. SiO_2_, the most widely used gate dielectric in microelectronics, possesses a relatively low dielectric constant (~3.9), a wide bandgap (~9 eV), and supports SOP modes capable of interacting with carriers in adjacent 2D layers. These phonon–carrier interactions, mediated primarily by the long-range Fröhlich mechanism, can markedly affect carrier mobility, scattering dynamics, and optical responses in TMDC monolayers, particularly under strong electric fields or elevated temperatures [17]. By contrast, hBN provides an atomically smooth, chemically inert interface with high thermal conductivity and a wide bandgap (~6 eV). Its low defect density helps preserve the intrinsic properties of TMDCs while reducing charge inhomogeneities and suppressing nonradiative recombination processes [18]. Thus, a detailed understanding of the interplay between substrate phonons and two-dimensional layers is essential for advancing charge transport and optical performance in TMDC-based heterostructures.

When integrated with insulating substrates such as SiO_2_ and hBN, platinum-based TMDCs form van der Waals heterostructures with markedly improved optoelectronic performance. For example, graphene/PtSe_2_/ultrathin SiO_2_/Si stacks display high carrier mobility, thickness-dependent bandgap transitions, and efficient broadband photodetection spanning the visible to infrared range [11]. Likewise, PtS_2_ thin films grown on SiO_2_ maintain robust semiconducting behavior and structural uniformity, making them suitable for field-effect transistors and diverse optoelectronic devices [15,16]. In contrast, hBN substrates offer distinct advantages due to their atomically smooth surface, wide bandgap, and chemical inertness. In PtSe_2_/hBN and PtS_2_/hBN heterostructures, the intrinsic properties of the active TMDC layers are better preserved, while substrate-induced disorder and charge-trapping effects are significantly mitigated [19,20]. As a result, such combinations yield enhanced electrical stability, superior optical performance, and reduced noise, which are crucial for applications in flexible electronics, broadband photodetectors, and high-performance spintronic systems.

Recent experimental and theoretical investigations have provided valuable insights into the structural and functional characteristics of PtSe_2_ and PtS_2_ on dielectric substrates such as SiO_2_ and hBN. Zhu et al. demonstrated the fabrication of high-performance graphene/PtSe_2_/ultrathin SiO_2_/Si photodetectors exhibiting broadband sensitivity from the visible to the infrared, together with ultrafast photoresponse. These attributes were attributed to the high carrier mobility and thickness-dependent tunable band structure of the PtSe_2_ layer [11]. On the hBN side, Kim et al. reported the molecular beam epitaxial growth of PtSe_2_ directly on hBN, achieving broadband and ultrafast photodetectors with enhanced photocurrent response and reduced defect-related scattering, a direct consequence of the atomically flat and chemically inert hBN surface [19]. Similarly, Li et al. fabricated few-layer PtS_2_ phototransistors on hBN, revealing high photoconductive gain, efficient carrier transport, and low-noise operation—key attributes for next-generation, low-power, and flexible optoelectronic platforms [20]. Collectively, these studies highlight the strong synergy between experimental and theoretical approaches in the design and optimization of Pt-based van der Waals heterostructures for advanced optoelectronic, sensing, and spintronic applications.

Although PtS_2_ and PtSe_2_ share comparable layered crystal structures, their chemical stability differs markedly owing to the distinct reactivity of sulfur and selenium. In both compounds, platinum predominantly adopts the +4 oxidation state, as verified by X-ray Photoelectron Spectroscopy. PtSe_2_ exhibits excellent stability under ambient and moderate thermal conditions, with minimal oxidation of selenium. By contrast, PtS_2_ is more prone to degradation: sulfur atoms readily oxidize to form surface sulfates, while platinum may undergo partial reduction even at relatively low temperatures. These stability differences are crucial when selecting materials for applications requiring thermal robustness, chemical durability, and stable interfacial properties [21,22].

Electron–phonon interactions at the interface between two-dimensional materials and polar substrates have attracted considerable attention because remote or SOPs can significantly influence carrier transport and energy relaxation. In supported two-dimensional systems, long-range Fröhlich-type coupling between charge carriers and polar substrate modes has been shown to limit carrier mobility and modify hot-carrier relaxation dynamics, particularly at elevated temperatures [23,24]. In transition-metal dichalcogenide monolayers, electron–phonon coupling also plays a crucial role in determining carrier transport and relaxation processes [25,26]. However, despite the considerable progress achieved in understanding electron–phonon interactions in supported two-dimensional materials, the combined influence of substrate-induced SOPs, polaronic hybridization, oscillator-strength redistribution, and Auger recombination in Pt-based group-X TMDC monolayers has not yet been systematically investigated within a unified theoretical framework. In this work, we address this issue by developing an analytical model that describes the interplay between these mechanisms in monolayer PtSe_2_ and PtS_2_ supported on polar dielectric substrates such as SiO_2_ and hBN.

The present study is motivated by several unresolved questions regarding carrier relaxation in supported Pt-based TMDC monolayers. In particular, it remains unclear how substrate-induced SOPs influence the polaronic spectrum, how this interaction leads to anticrossing behavior and oscillator-strength redistribution, and under what conditions Auger recombination becomes comparable to phonon-mediated relaxation processes. Addressing these questions is essential for understanding the dominant energy-dissipation mechanisms in supported PtSe_2_ and PtS_2_ heterostructures.

To further clarify these issues, it is important to assess the role of competing carrier relaxation mechanisms in supported Pt-based TMDC monolayers. While previous studies have examined substrate-induced SOP interactions in individual two-dimensional materials, a systematic comparison between different Pt-based transition-metal dichalcogenide monolayers and the role of competing carrier relaxation mechanisms remains largely unexplored. In particular, the interplay between substrate-induced SOPs and Auger recombination has not yet been investigated within a unified theoretical framework for PtSe_2_ and PtS_2_ monolayers. Addressing this gap, the present work provides a comparative analysis of these mechanisms in Pt-based TMDC monolayers supported on SiO_2_ and hBN substrates. This approach allows us to identify the relative importance of phonon-mediated and many-body relaxation processes and to clarify how the dielectric environment governs carrier relaxation dynamics and electron–phonon coupling in these systems.

In this work, we present a detailed theoretical study of electron–SOP interactions in ML PtS_2_ and PtSe_2_ supported on polar dielectric substrates, namely SiO_2_ and hBN. These long-range interactions, mediated by polarization fields induced in the substrate, strongly influence the optoelectronic behavior of two-dimensional materials. Our investigation begins with an analysis of the electronic band structure near the K-point, followed by the formulation of a Fröhlich coupling model to capture SOP-induced effects. We then examine the emergence of polaronic hybrid states and the associated Rabi-like energy splitting. Particular attention is devoted to evaluating the polaronic oscillator strength, which quantifies the modification of light–matter coupling in the presence of interfacial phonons. In addition, the dependence of the SOP-induced scattering rate on temperature and van der Waals separation distance is systematically analyzed, emphasizing the critical role of dielectric screening and interfacial proximity. Finally, we investigate the Auger recombination process in ML PtS_2_ and PtSe_2_ supported on SiO_2_ and hBN polar dielectric substrates. Collectively, these findings provide fundamental insight into substrate-mediated many-body interactions in ML PtS_2_ and PtSe_2_, offering design principles for the development of next-generation optoelectronic devices.

## 2. Lattice and Electronic Band Structure

**Figure 1:**
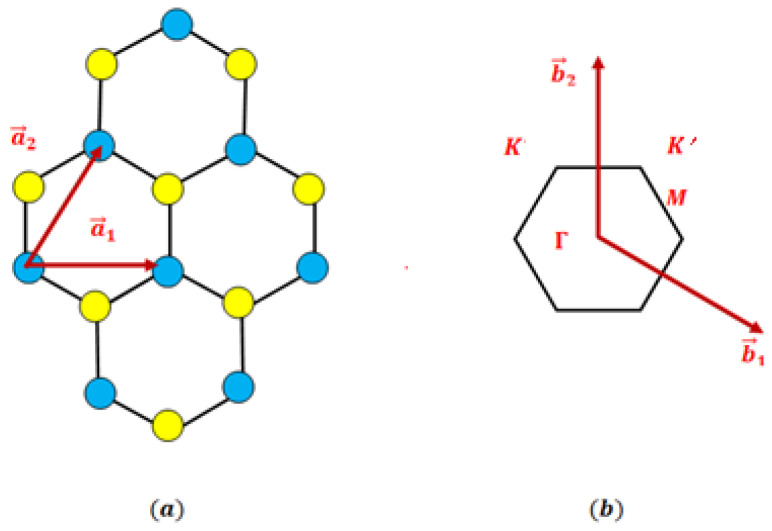
(**a**) The triangular Bravais lattice of monolayer PtS_2_. Blue and yellow spheres denote the platinum (Pt) atoms and sulfur (S) atoms, respectively. (**b**) The first Brillouin zone and high-symmetry points Γ, K, and M of PtS_2_ in reciprocal space of the triangular lattice. Its primitive lattice vectors are ${\vec{b}}_{1}$ and ${\vec{b}}_{2}$.

Monolayer PtS_2_ and PtSe_2_ belong to the group-10 TMDCs and crystallize in the 1T-phase structure. In this arrangement, a central layer of platinum (Pt) atoms is sandwiched between two layers of chalcogen atoms—sulfur (S) in PtS_2_ or selenium (Se) in PtSe_2_. The atoms form a hexagonal configuration in which the Pt atoms occupy a two-dimensional triangular Bravais lattice, while the chalcogen atoms are located in aligned positions above and below the Pt plane. The real-space lattice is defined by the primitive vectors ${\vec{a}}_{1}=a_{0}\left(1,0,0\right)$ and ${\vec{a}}_{2}=\frac{a_{0}}{2}\left(1,\sqrt{3},0\right)$, where $a_{0}$ denotes the in-plane lattice constant (see Figure 1a). In reciprocal space, the structure is also triangular, with reciprocal lattice vectors given by lattice ${\vec{b}}_{1}=\frac{4\pi }{\sqrt{3}a_{0}}\left(\frac{\sqrt{3}}{2},-\frac{1}{2},0\right)$ and ${\vec{b}}_{2}=\frac{4\pi }{\sqrt{3}a_{0}}\left(0,1,0\right)$ as illustrated in Figure 1b. The first Brillouin zone adopts a hexagonal geometry and contains the standard high-symmetry points: Γ at the zone center, K and K′ at the hexagonal corners, and M at the midpoints of the edges. These symmetry points govern the electronic, optical, and vibrational characteristics of PtS_2_ and PtSe_2_, playing a central role in band structure dispersion, excitonic behavior, and electron–phonon coupling phenomena.

The corresponding reciprocal lattice also exhibits a hexagonal geometry, with the first Brillouin zone containing high-symmetry points conventionally denoted as Γ, K, and M. These points are fundamental in determining the electronic band structure and are particularly relevant for analyzing band dispersion, optical transitions, and electron–phonon interactions. Unlike many group-6 TMDCs such as MoS_2_, monolayer PtSe_2_ and PtS_2_ possess an indirect bandgap, where the valence band maximum (VBM) is located at the Γ point and the conduction band minimum (CBM) lies near the M point. This distinctive band alignment strongly affects their optical absorption and charge transport characteristics, thereby influencing their suitability for specific optoelectronic and sensing applications. The coordinates of the principal high-symmetry points in reciprocal space are defined as follows:

$$\Gamma =\left(0,\;0\right),K=\left(\frac{2\pi }{3a_{0}},\;\frac{-2\pi }{\sqrt{3}a_{0}}\right),M=\left(\frac{\pi }{a_{0}},\;\frac{\pi }{\sqrt{3}a_{0}}\right)$$

In the present work, we employ the same theoretical framework, computational methodology, and governing equations as those extensively developed and discussed in our earlier studies [17,18,27,28,29,30,31,32]. The reader is referred to these works for full derivations and details of the implementation procedures.

For the analysis below, we use the notation $k=\left|\vec{k}\right|$ and $\left|K\right|=\left|\vec{\Gamma K}\right|=\frac{4\pi }{3\sqrt{3}a}$ to denote the magnitude of both wave vectors $\vec{k}$ and $\vec{\Gamma K}$, respectively, and we omit the vector notation for simplicity.

In order to describe the electronic states relevant to the scattering processes considered in this work, we employ a low-energy effective Hamiltonian for the electronic dispersion near the K and K′ valleys of the Brillouin zone. This approach leads to a Dirac-like energy dispersion in the vicinity of the band extrema. Such effective Hamiltonian descriptions are widely used in theoretical studies of carrier dynamics in transition-metal dichalcogenide monolayers, since the dominant scattering processes typically occur close to the valley minima. Effective models of this type have been successfully employed to describe the electronic structure and carrier dynamics of TMDC monolayers in numerous studies [33]. Since the scattering processes investigated in this work involve electronic states located in the vicinity of the K valleys, the Dirac-like approximation remains valid over the momentum range considered in the present calculations.

In the vicinity of the K valley, the conduction and valence band states with parallel spin $s=+\frac{1}{2}$ can be effectively described using a reduced 2 × 2 Hamiltonian formalism [33,34,35]. The corresponding energy dispersion obtained from this effective model takes the form of a Dirac-like spectrum [33,34,35]:

(1) $$\varepsilon_{\lambda ,k}=\lambda \varepsilon_{k}=\sqrt{{\left(\frac{E_{g}}{2}\right)}^{2}+\gamma^{2}k^{2}}$$

where λ=+ and λ=− label the conduction and valence bands, respectively. Here, γ is proportional to the interband momentum matrix element, and $E_{g}$ denotes the bandgap width of monolayer PtS_2_ and PtSe_2_. For computational feasibility, we assume idealized conditions in which the PtS_2_ and PtSe_2_ monolayers form uniform, defect-free interfaces with the underlying dielectric substrates. This assumption, while simplified, is commonly adopted in theoretical modeling and simulation to capture the essential physics without the complexity introduced by structural imperfections.

## 3. SOP Coupling Model

In this work, we investigate the interaction between electrons and SOPs in monolayer PtSe_2_ and PtS_2_ supported on SiO_2_ and hBN substrates within the framework of long-range Fröhlich coupling. This model provides a robust theoretical foundation for describing electron–SOP interactions in two-dimensional materials on polar substrates. Nevertheless, it involves several simplifying assumptions. Specifically, it is based on the Born–Oppenheimer approximation [36] and neglects short-range electron–phonon interactions, which may become relevant in non-polar environments [25]. It further assumes a constant effective electron mass and typically ignores nonlinear effects as well as multiphonon processes [37]. In addition, the influence of impurities, defects, and other types of structural disorder is not explicitly included [25,36,37,38]. Phonon dispersion is often approximated as linear, which may not hold across all phonon branches or substrate materials [39].

Within the present theoretical framework, the SOP modes are treated using the Fröhlich interaction model, which assumes that the relevant optical phonon modes are weakly dispersive near the interface. This approximation is commonly adopted in studies of electron–phonon interactions in supported two-dimensional systems because optical phonon modes in polar materials typically exhibit relatively small dispersion near the Brillouin-zone center. Although more complex phonon dispersions may slightly modify the quantitative values of the scattering rates, the approximation used here captures the dominant physical mechanisms governing the long-range interaction between charge carriers and SOPs in supported TMDC heterostructures.

Finally, the analysis generally considers a single dominant phonon mode, thereby neglecting possible contributions from additional modes.

For simplicity, the phonon spectrum is treated as isotropic, allowing phonons to be classified as either longitudinal or transverse. Within this framework, the electron–phonon interaction described by the Fröhlich Hamiltonian corresponds to a scattering process in which an electron with initial momentum $\vec{k}$ is scattered into a final state $\vec{k^{\prime }}=\vec{k+q}$ through the emission or absorption of a phonon.

While intrinsic electron–phonon coupling with longitudinal optical (LO) phonons near the K point is often regarded as a major scattering channel in free-standing monolayers of PtS_2_ and PtSe_2_, its relative contribution is substantially diminished in supported heterostructures. In these systems, scattering by remote SOPs originating from polar substrates becomes the dominant mechanism. This predominance, particularly under moderate thermal conditions (T < 300 K), can be justified by several physical arguments.

First, the Fröhlich interaction with SO phonons is intrinsically amplified in two-dimensional systems, owing to the confinement of electronic states and the characteristic 1/q dependence of the coupling strength at small momentum transfer [26]. Second, because the Fröhlich interaction is long-ranged and favors small-momentum scattering near the K point, short-range optical phonon interactions play a comparatively minor role. In contrast, substrate-induced SOPs couple efficiently to carriers through long-range polarization fields, and therefore, remain active in supported configurations. Third, the typical energy window of SOP modes (50–110 meV) ensures their thermal population at room temperature, enabling efficient single-phonon scattering while suppressing multiphonon contributions, which are exponentially unlikely under moderate temperatures [40].

Taken together, these considerations justify the exclusion of short-range, defect-assisted, and multiphonon processes in the present analysis. Their omission does not compromise the validity of the substrate-dependent scattering trends reported here but instead highlights the central role of remote SOPs in governing charge transport and optoelectronic performance in PtS_2_ and PtSe_2_ heterostructures.

Electron–SOP interactions intrinsic to the PtS_2_ and PtSe_2_ monolayers were not included because these materials do not support intrinsic surface polar modes. Monolayer PtSe_2_—and analogously PtS_2_—crystallizes in the centrosymmetric 1T phase, which preserves inversion symmetry and therefore lacks any out-of-plane dipole moment or infrared-active surface phonon modes [41]. This structural characteristic has been explicitly demonstrated by Cheon et al. [42], who showed that monolayer PtSe_2_ retains inversion symmetry and belongs to the centrosymmetric space group P-3m1. As a consequence, non-polar, centrosymmetric monolayers cannot generate macroscopic surface-polarization fields.

In contrast, genuine SOPs arise only in polar dielectrics, where the discontinuity between $\varepsilon_{0}$ and $\varepsilon_{\infty }$ produces long-range evanescent Fröhlich fields with a characteristic ∼1/q dependence at the interface, as established by Fischetti et al. [40] and by Hwang & Das Sarma [43]. Such fields cannot be generated in non-polar monolayers such as PtS_2_ and PtSe_2_. Accordingly, only the polar substrates (SiO_2_ and hBN) host true SO phonon modes that couple efficiently to carriers and dominate long-wavelength electron scattering in supported heterostructures.

Intrinsic LO/TO phonons of PtS_2_ and PtSe_2_ are short-range intralayer modes and do not mediate long-range Fröhlich interactions; their coupling to electronic states near the K valleys is known to be weak in the relevant small-q regime [26]. While intrinsic LO/TO phonons may contribute more significantly in other regions of the Brillouin zone or in suspended monolayers, they remain subdominant in supported samples. The adopted treatment therefore captures the dominant substrate-dependent electron–phonon interactions that dictate carrier mobility and hot-carrier relaxation in PtS_2_/SiO_2_, PtSe_2_/SiO_2_, PtS_2_/hBN, and PtSe_2_/hBN heterostructures.

In order to describe the interaction between charge carriers in the monolayer and SOPs originating from the polar substrate, we adopt a theoretical framework based on the long-range Fröhlich coupling model. The interaction between charge carriers in the monolayer and polar phonons of the dielectric substrate is described using the Fröhlich coupling model. This model provides a well-established theoretical framework for describing long-range electron–phonon interactions in polar materials. In supported two-dimensional systems, SOPs generated in the polar substrate produce electric fields that penetrate into the adjacent monolayer, leading to a long-range Coulomb interaction between carriers and substrate phonons. The Fröhlich model is therefore particularly suitable for describing remote phonon scattering in supported two-dimensional materials. In addition, this approach allows an analytical description of the electron–phonon interaction while capturing the essential physics governing carrier relaxation and scattering processes in polar environments. Similar theoretical treatments have been widely used in the literature to investigate substrate-induced electron–phonon coupling in two-dimensional systems [23,24,26]. Within this framework, the electron–phonon interaction can be described using the Fröhlich Hamiltonian given below.

Within this framework, the electron–phonon interaction is described by the Fröhlich Hamiltonian for long-range electron–phonon coupling, given below [44]:

(2) $$H=\sum_{q,\nu }\hbar \omega_{\nu }a_{q}^{+}a_{q}+\sum_{q,\nu }M_{q,\nu }\left(a_{-q}^{+}{+\;a}_{q}\right)e^{-iq\;r}$$

The interaction between charge carriers in monolayer PtSe_2_ and PtS_2_ and SOPs is represented by the second term in Equation (2). Here, $a_{q}^{+}$, $a_{q}$ denote the phonon creation and annihilation operators, respectively, for a mode characterized by wave vector q, while $\omega_{\nu }$ corresponds to the phonon frequency, with ν=1, 2 labeling the two relevant phonon branches. The coupling matrix element $M_{q,\;\nu }$ in the Fröhlich Hamiltonian quantifies the interaction strength between an electron in the monolayer and a SOP mode of the polar substrate. It is expressed as follows [45,46,47]:

(3) $$V_{SOP}{=M}_{q,\;SO}=\left|\vec{k}-\vec{k+q}\right|\sqrt{\frac{e^{2}\;\frac{\hbar \omega_{SO,\nu }}{2\pi }\left(\frac{1}{\varepsilon_{\infty }+\varepsilon_{env}}-\frac{1}{\varepsilon_{0}+\varepsilon_{env}}\right)}{2NAq}}e^{-qd}$$

where A is the area of the monolayer, d is the vertical distance between the monolayer and the substrate, $\varepsilon_{0}$ and $\varepsilon_{\infty }$ denote the static and high-frequency dielectric constants of the substrate associated with, $\hbar \omega_{SO,\nu }$ corresponds to the energy of the SOPs, with ν=1, 2. denoting the two phonon branches. The screening of the Coulomb interaction by the surrounding polar dielectric environment is accounted for using the parameter $\varepsilon_{env}.$

For clarity and reproducibility, the principal parameter sources and modeling assumptions used in this work are summarized here. The dielectric constants and optical phonon parameters of the SiO_2_ and hBN substrates employed in the present calculations are listed in Table 1. These parameters were taken from previously reported experimental and theoretical studies cited in the manuscript. The electronic band gaps and effective electron masses used in the calculations are summarized in Table 2, and were adopted from first-principles density functional theory (DFT) studies reported in the literature.

The present analytical treatment assumes long-range Fröhlich-type electron–phonon coupling between charge carriers in the monolayer and polar phonon modes originating from the substrate. Several simplifying approximations are adopted in order to maintain analytical tractability. In particular, short-range defect scattering, disorder effects, multiphonon processes, and plasmon–phonon hybridization are neglected. The monolayer is treated as an electrostatically infinitesimal two-dimensional sheet, and only the coupling between charge carriers in the monolayer and substrate-induced SOPs is considered. These approximations follow standard approaches widely used in the literature and define the physical scope of the present analytical model.

The detailed parameter values, their literature sources, and the analytical expressions used to describe the electron–phonon coupling are presented in the following sections and summarized in Table 1 and Table 2, while the corresponding modeling assumptions and approximations are discussed together with their supporting references in the subsequent text.

For SiO_2_, the dielectric constants and optical phonon energies were taken from experimental optical and infrared measurements reported by Fischetti et al. [40]. The monolayer–substrate separation d was estimated theoretically using an electrostatic interface model [40].

For hBN, dielectric and phonon parameters were obtained from experimental infrared spectroscopy data reported by Geick et al. [48], while the interface distance was theoretically determined based on van der Waals interactions [48].

In both cases, the Fröhlich coupling constant $F_{\nu }^{2}$ was computed following the analytical framework developed by Fratini et al. [23], incorporating the substrate-specific dielectric environment and phonon characteristics:

(4) $$F_{\nu }^{2}=\frac{\hbar \omega_{SO,\nu }}{2\pi }\left(\frac{1}{\varepsilon_{\infty }+\varepsilon_{env}}-\frac{1}{\varepsilon_{0}+\varepsilon_{env}}\right)$$

The SOP energies are derived from the bulk LO phonon modes [49]:

(5) $$\hbar \omega_{SO}=\hbar \omega_{LO}{\left(\frac{1+\frac{1}{\varepsilon_{0}}}{1+\frac{1}{\varepsilon_{\infty }}}\right)}^{\frac{1}{2}}$$

where $\hbar \omega_{LO}$ denotes the energy of the bulk LO phonon. The screening of the Coulomb interaction by the surrounding polar dielectric environment is incorporated through the parameter $\varepsilon_{env}$. Due to the weak screening of the out-of-plane electric field in monolayer PtSe_2_ and PtS_2_, $\varepsilon_{env}$ is taken to be unity [50].

The electronic band gaps and effective electron masses employed in this study were obtained from first-principle DFT calculations. Bandgap values were adopted from Zhao et al. [12], while effective electron masses were extracted from the theoretical mobility analysis of Zhang et al. [51], which is based on ab initio band structures and deformation potential theory. A summary of these parameters is provided in Table 2.

**Table 2:** Band gap and effective electron mass of ML PtS_2_ or PtSe_2_.

	${PtS}_{2}$	${PtSe}_{2}$
$E_{g}\left(\mathrm{eV}\right)$ [12]	1.76	1.37
$m^{*}\left(m_{0}\right)$ [51]	0.26	0.21

In the present study, we focus on supported monolayer systems, specifically PtS_2_/SiO_2_, PtS_2_/hBN, PtSe_2_/SiO_2_, and PtSe_2_/hBN, where SOPs originate from the polar nature of the substrate and couple to the monolayer via long-range electrostatic fields. These interactions are described using a simplified two-dimensional Fröhlich-type model [17,18,27,28,29,30,31,32,52], in which SOPs are treated as substrate-confined vibrational modes that interact with charge carriers in the adjacent monolayer.

Several approximations are adopted in this approach. The monolayer is considered an electrostatically infinitesimal 2D sheet; its intrinsic dielectric response is neglected, and the coupling between substrate SOPs and the monolayer’s intrinsic LO phonons is ignored. The system is reduced to a single 2D/substrate interface, and effects such as finite thickness, plasmon–phonon hybridization, and multi-phonon scattering are not included. While these simplifications are standard in the literature and allow analytical tractability, more rigorous modeling—such as solving Poisson’s equation with realistic layered dielectric boundary conditions—would be required to capture hybridization effects and spatially inhomogeneous screening. This represents an important avenue for future investigations.

From a quantitative perspective, the coupling matrix element describing the interaction with intrinsic LO phonons near the K point can be written as follows. The quantity $V_{LO}$ denotes the interaction potential associated with the LO phonon mode [52]:

(6) $$V_{LO}{=M}_{q,\;LO}=\sqrt{\frac{D_{op}^{2}\;\hbar }{4NM_{c}\omega_{K}}\left(1-\cos\left(\theta_{k}-\theta_{k+q}\right)\right)}$$

Here, $\omega_{K}$ is the LO phonon frequency near the K point, $M_{c}$ is the effective mass of a PtS_2_ (or PtSe_2_) unit, and ${D_{op}=2D}_{\Gamma }^{2}$ is an effective electron–optical phonon coupling constant. $\theta_{k}$ is the angle associated with the direction of the wave vector $\vec{k}$.

Although the intrinsic LO phonon coupling is generally stronger, the interaction with SOPs—quantified by the matrix element ${\left|M_{q,\;SO}\right|}^{2}$—can be appreciable in specific dielectric environments. In particular, the relatively low SOP energy of SiO_2_ (~58.9 meV) combined with its pronounced polar character enhances the long-range Fröhlich-type interaction, especially for small van der Waals separations d, as the coupling strength scales with $e^{-qd}$. In contrast, hBN exhibits weaker polarity; nevertheless, its high-energy SO modes (~101.7 meV) can still influence electron dynamics under conditions of low carrier density and limited dielectric screening.

In summary, although ${\left|M_{q,\;SO}\right|}^{2}$ is generally smaller than ${\left|M_{q,\;LO}\right|}^{2}$, the interaction with substrate-induced SOPs makes a significant contribution to carrier scattering and optical transitions in supported monolayers. Consequently, its inclusion is essential for a complete description of electron–phonon interactions in PtS_2_ and PtSe_2_ heterostructures. The long-range Fröhlich model, despite its inherent approximations, offers a robust analytical framework for estimating these interactions and continues to be widely employed in studies of two-dimensional semiconductors on polar substrates.

Building upon this theoretical foundation, we now present the analytical form of the coupling between electrons and SOPs. In polar substrates, SOPs generate an electric field that interacts with electrons in the adjacent monolayer PtSe_2_ or PtS_2_. The strength of this coupling can be expressed as:

(7) $${W=\sum_{\vec{q}}\left|\left\langle \psi_{k}|V_{SOP}|\psi_{k+q}\right\rangle \right|}^{2}=\frac{NA}{{\left(2\pi \right)}^{2}}\iint \frac{1-\cos\left(\theta_{k}-\theta_{k+q}\right)}{2}\frac{4\pi^{2}e^{2}F_{\nu }^{2}}{NAq}e^{-2qd}qdqd\theta_{q}$$

The summation is performed over a single spin orientation and a single valley. Here, $A=\frac{\sqrt{3\;}}{2}a^{2}$ denotes the area of the two-atom unit cell, with a representing the lattice constant. The electronic states $\left|\psi_{k}\rangle \right.$ and $\left|\psi_{k+q}\rangle \right.$ correspond to the initial and final states involved in the scattering process, where an electron transitions from $\vec{k}$ to $\vec{k^{\prime }}=\vec{k+q}$ via the emission or absorption of a phonon.

In this work, we employ the same theoretical approach as detailed in our previous studies [17,18,27,28,29,30,31,32]. To analyze electron–SOP interactions in monolayer PtSe_2_ and PtS_2_, we focus on the electronic states $\left|\psi_{k}\rangle \right.$ and $\left|\psi_{k+q}\rangle \right.$, associated with energies $E_{k}=\varepsilon_{k}$ and $E_{k+q}=\varepsilon_{k+q}$, respectively. Furthermore, an effective 2 × 2 Hamiltonian is employed to describe the conduction and valence band states with parallel spin projections $s=\pm \frac{1}{2}$, in the vicinity of the $K(K^{\prime })$ valley of the hexagonal Brillouin zone.

For the numerical implementation of the developed model, a dedicated approach was adopted. The Fröhlich-type electron–SOP coupling equations were solved using an in-house Fortran-based computational code. This code performs a self-consistent numerical solution of the momentum- and energy-resolved equations derived from the interaction Hamiltonian, enabling accurate evaluation of polaronic properties, including oscillator strengths and scattering rates, in supported monolayer PtS_2_ and PtSe_2_ systems.

## 4. Polaronic State Formation and Rabi Splitting

The polaronic state space is formulated as the tensor product of the electronic and phononic Hilbert subspaces. Consequently, the complete system is represented in terms of a new basis set, denoted as polaronic states, which incorporate both the bare electronic configurations and their coupling with single-phonon excitations. The detailed construction of this basis, together with the formal development of the method, is reported in our earlier studies [17,18,27,28,29,30,31,32].

The corresponding polaronic electron energies $E_{\pm }^{e}$ associated with the states $\left|\psi_{\pm }\rangle \right.$ in monolayer PtSe_2_ and PtS_2_ supported on polar substrates are given by the following expression [17,18,27,28,29,30,31,32].

(8) $$E_{\pm }^{e}=\frac{1}{2}\left(E_{k+q}+E_{k}+\hbar \omega_{LO}\right)\pm \sqrt{{\left[\frac{1}{2}\left(E_{k+q}-E_{k}+\hbar \omega_{LO}\right)\right]}^{2}+\frac{NA}{{\left(2\pi \right)}^{2}}\iint \frac{1-\cos\left(\theta_{k}-\theta_{q}\right)}{2}\frac{4\pi^{2}e^{2}F_{\nu }^{2}}{NAq}e^{-2qd}qdqd\theta_{q}}$$

Here, $E_{k}$ and $E_{k+q}$ are the electron energies associated with the electronic states $\left|\psi_{k}\rangle \right.$ and $\left|\psi_{k+q}\rangle \right.$ respectively.

**Figure 2:**
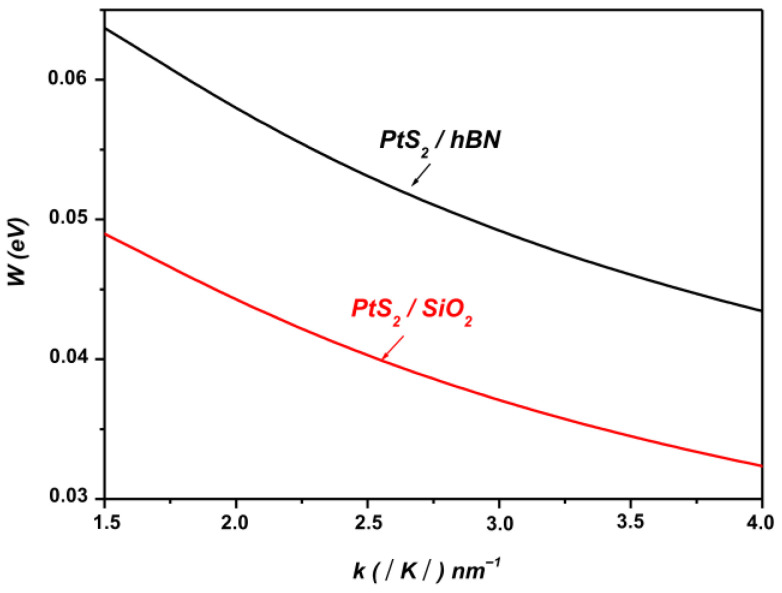
The strength of the SOP coupling between the electronic states $\left|\psi_{k}\rangle \right.$ and $\left|\psi_{k+q}\rangle \right.$ versus the wave vector k in ML PtS_2_ on SiO_2_ and hBN polar substrates. $\left(\left|K\right|=\frac{4\pi }{3\sqrt{3}a}\right)$, a is the lattice constant.

Figure 2 depicts the variation in the SOP coupling strength between the electronic states $\left|\psi_{k}\rangle \right.$ and $\left|\psi_{k+q}\rangle \right.$ as a function of the wave vector k in monolayer PtS_2_ supported on SiO_2_ and hBN polar substrates. The figure clearly demonstrates that the coupling with SOPs is highly sensitive to the nature of the underlying polar substrate.

**Figure 3:**
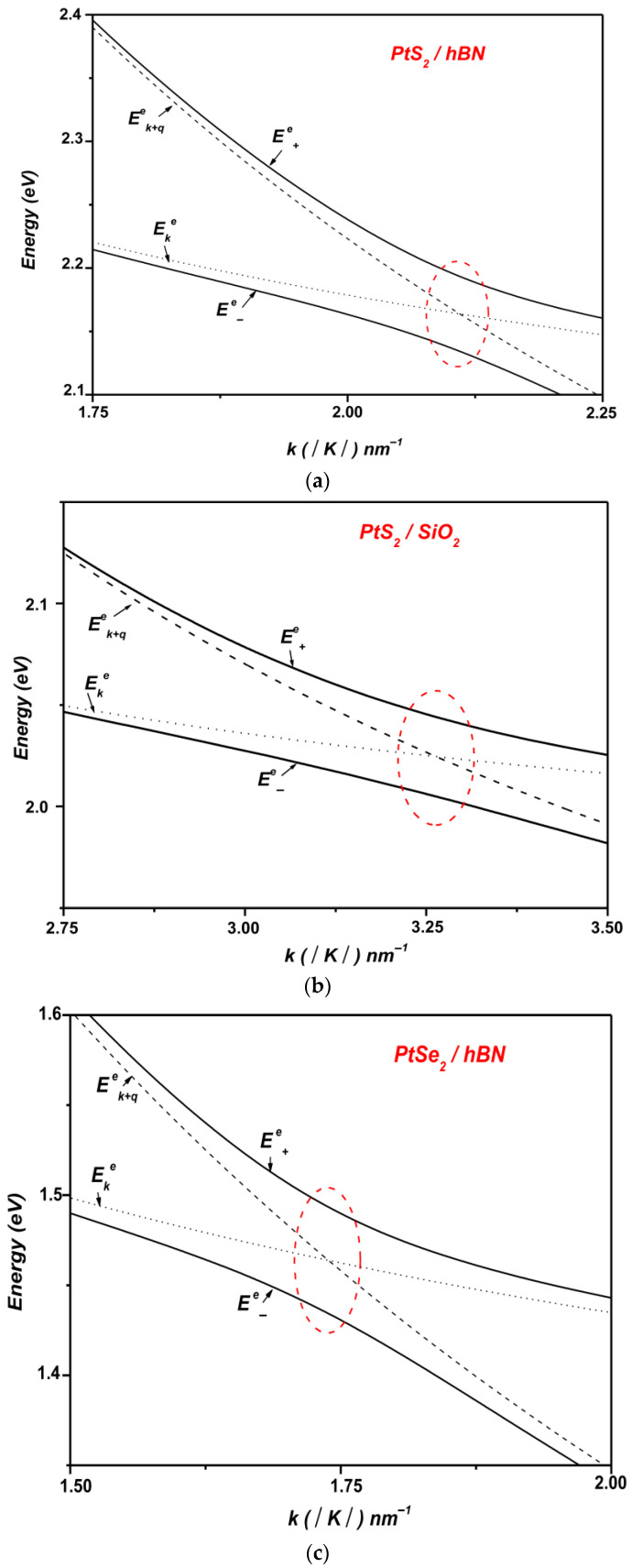

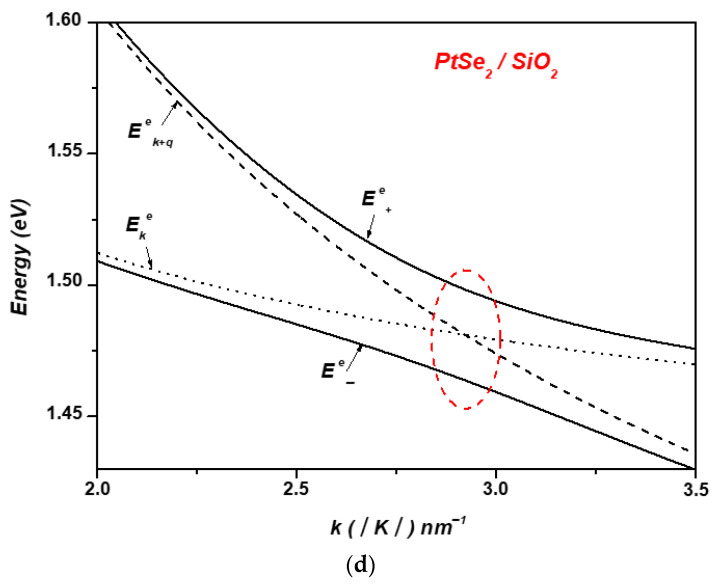
Polaron electron energies versus the wave vector k for monolayer PtS_2_ and PtSe_2_ supported on polar substrates: (**a**) PtS_2_/hBN, (**b**) PtS_2_/SiO_2_, (**c**) PtSe_2_/hBN, (**d**) PtSe_2_/SiO_2_. $\left(\left|K\right|=\frac{4\pi }{3\sqrt{3}a}\right),\;a$ is the lattice constant.

Figure 3 presents the polaronic electron energy dispersion as a function of the wave vector k for monolayer PtS_2_ and PtSe_2_ supported on polar substrates, namely SiO_2_ and hBN. For comparison, the corresponding non-interacting electronic states $\left|\psi_{k+q},\;0q\rangle \right.$ and $\left|\psi_{k},\;1q\rangle \right.$ are shown together with the interacting dispersion. In the case of PtS_2_, the non-interacting energy branches intersect at approximately $k\sim 3.26\;{nm}^{-1}$ on SiO_2_ and $k\sim 2.1\;{nm}^{-1}$ on hBN. These intersections reflect a resonant condition in which the energy separation between the electronic states matches the optical phonon energy $\hbar \omega_{LO}$, equal to 62.5 meV for SiO_2_ and 103.7 meV for hBN. Under such resonances, pronounced anticrossings emerge in the interacting spectrum, replacing the bare-level crossings and providing clear evidence of strong electron–phonon coupling. The corresponding Rabi splittings reach about 36 meV on SiO_2_ and 57 meV on hBN.

A comparable behavior is found for PtSe_2_, where the non-interacting states intersect at approximately $k\sim 2.92\;{nm}^{-1}$ on SiO_2_ and $k\sim 1.75\;{nm}^{-1}$ on hBN. In both cases, the condition for resonant coupling is satisfied, as the energy separation between the states coincides with the respective phonon energies. The strong coupling regime is evidenced by anticrossings, yielding Rabi splittings of about 40 meV on SiO_2_ and 60 meV on hBN. As shown in Figure 3, the Rabi splitting is significantly larger on hBN than on SiO_2_, highlighting the enhanced strength of the electron–phonon interaction in hBN-supported configurations.

A quantitative comparison between the two substrates highlights the stronger electron–phonon hybridization in the hBN-supported structures. For monolayer PtS_2_, the calculated Rabi splitting increases from approximately 36 meV on SiO_2_ to about 57 meV on hBN. Similarly, for monolayer PtSe_2_ the Rabi splitting increases from about 40 meV on SiO_2_ to about 60 meV on hBN. This systematic increase indicates that the dielectric environment of the substrate strongly influences the strength of the electron–phonon interaction and the resulting anticrossing gaps in supported TMDC monolayers.

In polar substrates, SOPs generate electric fields that penetrate the adjacent monolayer, where they couple directly with the electronic states of PtSe_2_ and PtS_2_ through dipole interactions. This coupling substantially enhances the electron–phonon interaction strength, leading to a renormalization of the electronic spectrum. Consequently, the energy separation between hybridized polaronic states increases, giving rise to an enhanced Rabi splitting.

The difference in Rabi splitting observed between SiO_2_ and hBN substrates (Figure 3) originates from their distinct surface phonon modes, dielectric responses, and phonon energy scales. In particular, hBN possesses a higher static dielectric constant compared to SiO_2_, which results in stronger interfacial electric fields associated with its surface phonons. This enhanced field strength promotes a more efficient coupling with the electronic states of monolayer PtSe_2_ and PtS_2_, thereby amplifying the electron–phonon interaction and producing a larger Rabi splitting.

In contrast, SiO_2_, owing to its relatively lower dielectric constant, produces weaker electric fields associated with its SOPs. Consequently, the coupling between these phonons and the electronic states in PtSe_2_ and PtS_2_ monolayers is reduced, leading to a smaller Rabi splitting. This comparison underscores the pivotal role of the polar substrate in governing the strength of the electron–phonon interaction, and thereby in modulating the optical response and performance of PtSe_2_- and PtS_2_-based optoelectronic devices.

At the anticrossing points, the electronic wave functions become hybridized, thereby allowing multiple transition pathways such as $E_{k}\to E_{\pm }^{e}$, $E_{k}\to E_{k}+\;\hbar \omega_{LO}$ and $E_{k}\to E_{k+q}$. This behavior demonstrates that the electron–SOP interaction cannot be adequately described within the weak-coupling regime. Instead, the strong-coupling condition leads to a Rabi splitting of the electronic levels. Theoretical analysis thus confirms the occurrence of resonant coupling between electronic subbands and surface vibrational modes in monolayer TMDCs supported on polar substrates.

The resulting hybridized states, or polarons, are given by:

(9) $$\left|\psi_{\pm }\rangle \right.=\alpha_{\pm }\left|\psi_{k+q},\;0q\rangle \right.+\;\beta_{\pm }\left|\psi_{k},\;1q\rangle \right.$$

The weights of the electronic component $\alpha_{\pm }$ and the one-phonon component $\beta_{\pm }$ of the polaronic states ± vary with the corresponding polaron energies $E_{\pm }^{e}$. The explicit expressions describing these dependencies are provided in our earlier works [17,18,27,28,29,30,31,32].

**Figure 4:**
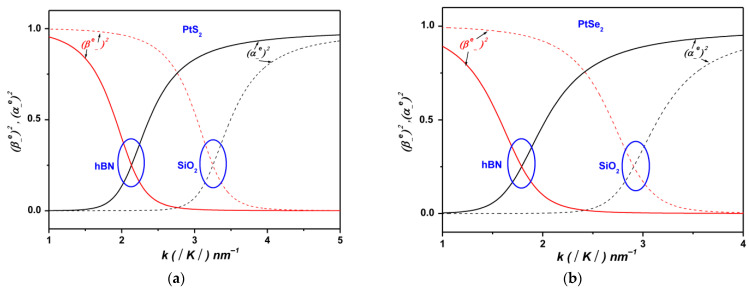
The weight of the electronic components and the one-phonon components of the lower polaron state $\left|\psi_{-}\rangle \right.$ versus the wave vector k,$\left(\left|K\right|=\frac{4\pi }{3\sqrt{3}a}\right)$, a is the lattice constant. (**a**) in PtS_2_/hBN and PtS_2_/SiO_2_ interfaces, (**b**) in PtSe_2_/hBN and PtSe_2_/SiO_2_ interfaces.

Figure 4 displays the variation in the electronic and one-phonon component weights of the lower polaronic state $\left|\psi_{-}\rangle \right.$ in monolayer PtS_2_ and PtSe_2_ supported on SiO_2_ and hBN substrates, as a function of the wave vector k. For ML PtS_2_ on hBN, the one-phonon component of $\left|\psi_{-}\rangle \right.$ becomes dominant near $k\sim 2.1\;{nm}^{-1}$, where its weight significantly surpasses that of the electronic component $\left(\beta_{\pm }\gg \alpha_{\pm }\right)$. This result highlights the pronounced role of SOPs at the PtS_2_/hBN interface, which promotes resonant coupling between the non-interacting states $\left|\psi_{k},\;1q\rangle \right.$ and $\left|\psi_{k+q},\;0q\rangle \right.$, thereby driving polaron formation [29,30,31,32,33,34,35,36]. Comparable trends are observed for the other configurations involving monolayer PtS_2_ and PtSe_2_ on both SiO_2_ and hBN (see Figure 4).

## 5. Polaronic Oscillator Strength

In the following section, we provide a theoretical analysis of the polaronic oscillator strength (OS), which serves as a key parameter for characterizing light–matter interactions in these systems. Drawing an analogy with interband transitions in quantum dots, we evaluate the OS for interband transitions in monolayer PtS_2_ and PtSe_2_ supported on polar substrates. In the strong-confinement regime, the OS is directly determined by the spatial overlap between polaronic states and is expressed through the squared modulus of the overlap integral, ${\left|\left(\psi_{-}\right)\right|}^{2}$, according to the following relation [24,53]:

(10) $$f_{Osc}=\frac{{\left|\left(\psi_{-}\right)\right|}^{2}E_{p}}{2\left(E_{g}+E_{-}\right)}$$

Here, $E_{P}$ denotes the Kane energy, while $\left(E_{g}+E_{-}\right)$ represents the emission energy associated with a single optical phonon in monolayer PtS_2_ and PtSe_2_ supported on polar substrates. In this context, $E_{-}$ corresponds to the energy of the lower polaronic electron branch, $\hbar \omega_{LO}$ is the energy of the emitted optical phonon, and $E_{g}$ denotes the electronic band gap of monolayer PtS_2_ and PtSe_2_. The oscillator strength has been evaluated for the lower polaronic state $\left|\psi_{-}\rangle \right.$, which emerges as a coherent superposition of the basis states $\left|\psi_{k+q},\;0q\rangle \right.$ and $\left|\psi_{k},\;1q\rangle \right.$.

**Figure 5:**
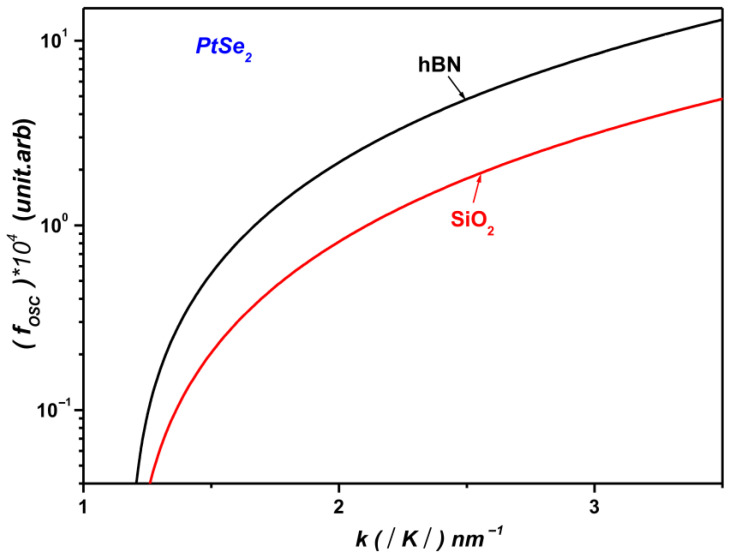
The polaronic OS of $Pt{Se}_{2}$ on the $SiO_{2}$ and *h*BN polar substrates vs. the wave vector, $\left(\left|K\right|=\frac{4\pi }{3\sqrt{3}a}\right)$, a is the lattice constant.

Figure 5 displays the calculated polaronic OS for monolayer PtSe_2_ on SiO_2_ and hBN substrates as a function of the wave vector k. The analysis reveals that the polaronic OS is strongly influenced by the optical phonon modes of the dielectric environment. This sensitivity arises from the dependence of the phonon emission energy, $E_{PL}$, on both the electronic band structure and the substrate’s phonon characteristics. Specifically, the emission energy is expressed as $E_{PL}=\left(\hbar \omega_{LO}=E_{g}+E_{-}\right)$, where $E_{g}$ is the band gap and $E_{-}$ is the lower polaron energy. Consequently, the highest oscillator strength is obtained for the substrate with the largest LO phonon energy, confirming the relation $E_{PL}=\left(\;\hbar \omega_{LO}\right)$.

Near the anticrossing region, the electronic and phonon-assisted states become strongly hybridized, leading to a redistribution of oscillator strength between the hybrid polaronic branches. Away from resonance, the OS remains primarily associated with the bare electronic state, whereas near the anticrossing wave vector, the hybridization induces a significant transfer of spectral weight between the two branches.

Applying the same theoretical framework, we find that the polaronic oscillator strength in monolayer PtS_2_ is significantly enhanced when the material is supported on an hBN substrate compared with SiO_2_. This enhancement is primarily attributed to the stronger polarization field associated with the SOPs of hBN, which enables more efficient coupling with the interfacial electronic states. These findings underscore the critical role of the substrate’s dielectric properties in modulating electron–phonon interactions in two-dimensional systems.

This behavior can be explained by the higher dielectric constant of hBN and its larger LO phonon energy relative to SiO_2_ $\left(\hbar \omega_{LO}\;\left(hBN\right)=103.7\;\mathrm{meV}\;>\;\hbar \omega_{LO}\;\left(SiO_{2}\right)=62.5\;\mathrm{meV}\right)$ (see Table 1). As a result, the polarization field induced at the monolayer–substrate interface is stronger for hBN, leading to a more pronounced polaronic optical response in both PtS_2_ and PtSe_2_ supported on hBN.

**Figure 6:**
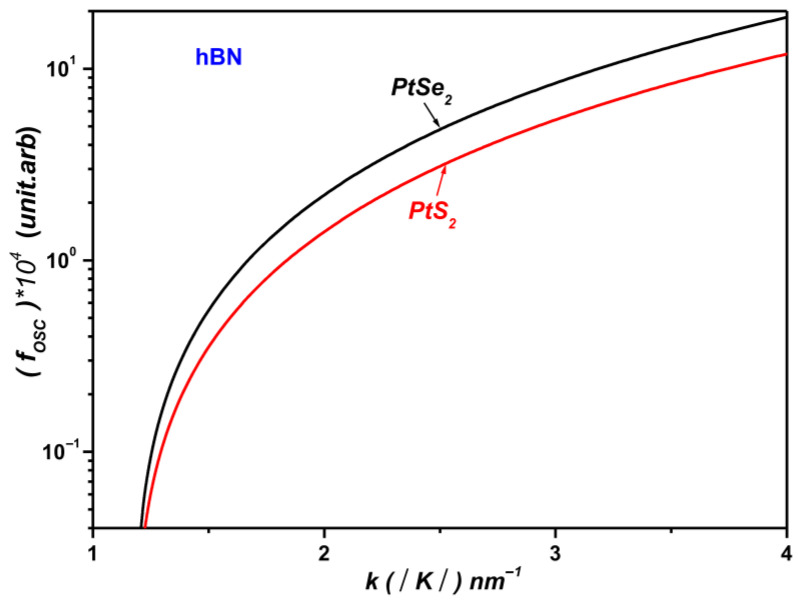
The polaronic OS in the ML $PtSe_{2}$ and ${PtS}_{2}$ on hBN polar substrate versus the wave vector k. $\left(\left|K\right|=\frac{4\pi }{3\sqrt{3}a}\right)$, a is the lattice constant.

Furthermore, as illustrated in Figure 6, monolayer PtSe_2_ exhibits a markedly stronger OS than monolayer PtS_2_. This enhancement arises from more efficient coupling between the SOPs of the polar substrate and the electronic states of PtSe_2_ near the interface, highlighting the material’s increased sensitivity to interfacial phonon-mediated interactions.

This behavior can be attributed to the differences in the effective electron masses of the two materials. In monolayer PtS_2_, the relatively heavier electrons lead to a reduced polaronic oscillator strength near the monolayer–dielectric interface. Conversely, the lighter electrons in monolayer PtSe_2_ result in a substantial enhancement of the OS, particularly under conditions of strong confinement.

## 6. SOP-Induced Scattering Rate

We now turn to the temperature dependence of the polaronic scattering rate induced by SOPs. The SOP scattering rate, which characterizes the rate of momentum relaxation for polarons interacting with interfacial phonons, is expressed as follows [17,18,27,28,29,30,31,32,52]:

(11) $$\begin{array}{l} \frac{1}{\tau_{Polaron}}=\frac{2\pi }{\hbar }\frac{NA}{{\left(2\pi \right)}^{2}}\iint \left[\frac{1-\cos\left(\theta_{k}-\theta_{k+q}\right)}{2}\frac{4\pi^{2}e^{2}F_{\nu }^{2}}{N\frac{\sqrt{3}}{2}a^{2}q}e^{-2qd}\right]\left[1-\cos\left(\theta_{k}-\theta_{k+q}\right)\right]\times \{N_{q}\delta \left(E_{k}-E_{k+q}+\hbar \omega_{q}\right)+\\ \left(N_{q}+1\right)\delta \left(E_{k}-E_{k+q}-\omega_{q}\right)\}qdqd\theta \end{array}$$

Here, $N_{q}$ represents the Bose–Einstein occupation number of the phonons, and $\theta_{k}$ denotes the angle corresponding to the direction of the wave vector $\vec{k}$.

**Figure 7:**
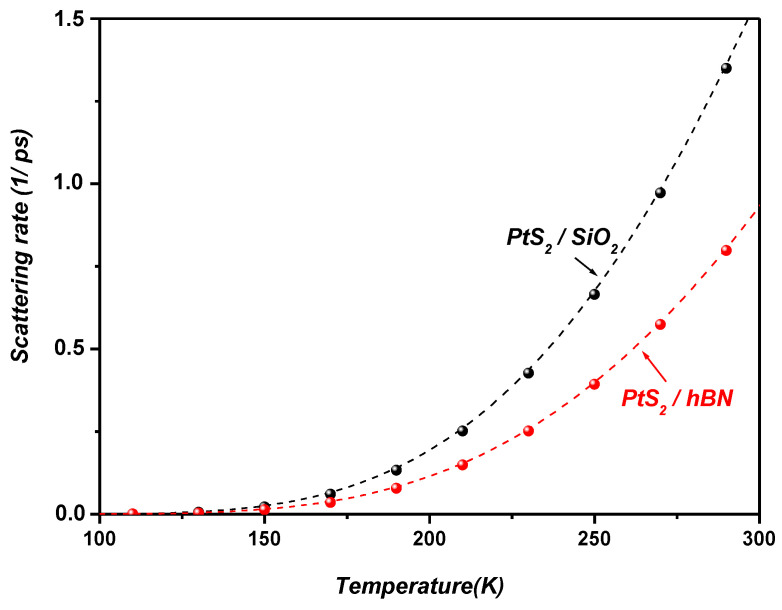
The temperature dependence of the SOP scattering rate in the ML $PtS_{2}$ on the $SiO_{2}$ and *h*BN dielectric polar substrates.

Figure 7 illustrates the temperature dependence of the SOP scattering rate in monolayer PtS_2_ supported on SiO_2_ and hBN substrates. The scattering rate increases monotonically with temperature as a direct consequence of the enhanced phonon population governed by the Bose–Einstein distribution. Across the entire temperature range, PtS_2_ exhibits a consistently higher SOP scattering rate on SiO_2_ than on hBN. This behavior is attributed to the lower SOP energies and the larger dielectric screening contrast ${(\varepsilon }_{0}-\varepsilon_{\infty })$ of SiO_2_, both of which strengthen the interfacial Fröhlich coupling and thereby enhance electron–phonon scattering compared to hBN. These findings highlight the crucial role of substrate-dependent dielectric and vibrational properties in governing carrier relaxation dynamics in supported two-dimensional materials.

**Figure 8:**
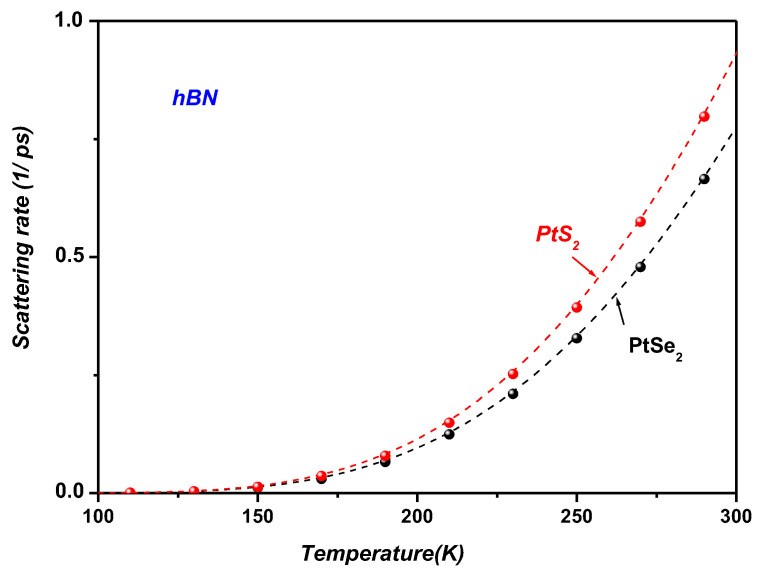
The temperature dependence of the SOP scattering rate in the ML $PtS_{2}$ and $Pt{Se}_{2}$ on *h*BN dielectric polar substrate.

Figure 8 shows that PtS_2_ exhibits a higher SOP scattering rate than PtSe_2_ when supported on hBN. This behavior can be attributed to the larger electron effective mass in PtS_2_ (0.26 m_0_ vs. 0.21 m_0_ for PtSe_2_; see Table 2), which enhances the Fröhlich-type electron–phonon coupling strength. Within the general theoretical framework for two-dimensional polar interactions [25], the SOP scattering rate scales with the carrier effective mass, leading heavier carriers to interact more strongly with substrate-induced SOPs. As a result, PtS_2_ experiences systematically stronger SOP scattering than PtSe_2_ across the entire temperature range.

## 7. Impact of van der Waals Separation

**Figure 9:**
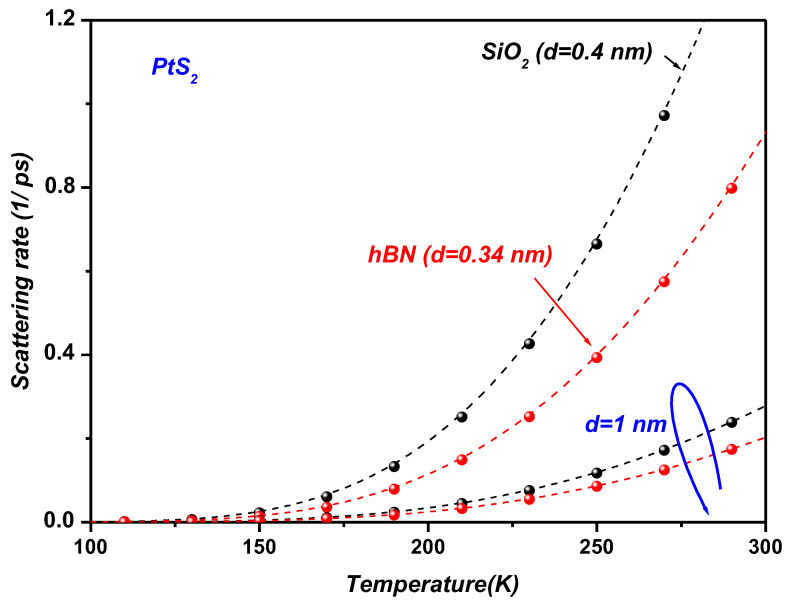
The temperature dependence of the SOP scattering rate in the ML $PtS_{2}$ on the $SiO_{2}$ and *h*BN dielectric polar substrates, for various van vdW separation distances.

We now examine the effect of van der Waals separation on SOP-induced scattering in monolayer PtS_2_ and PtSe_2_ supported on polar substrates, specifically SiO_2_ and hBN (Figure 9). At typical interface distances (d = 0.4 nm for SiO_2_, d = 0.34 nm for hBN, as reported in Table 1), strong electron–phonon coupling occurs due to the close proximity of the 2D layer to the SOP field. However, when the van der Waals distance increases to d = 1.0 nm, the SOP-induced scattering rate decreases dramatically—by more than an order of magnitude—because of the exponential decay of the electric field away from the substrate. This reduction enhances carrier mobility and improves the optoelectronic response. These findings highlight the critical role of interfacial spacing as a tunable parameter for controlling electron–phonon interactions in two-dimensional materials, with direct implications for the design of next-generation optoelectronic devices.

The observed reduction in the scattering rate with increasing interfacial distance d (from 0.34 nm to 1.0 nm) reflects the exponential weakening of the long-range Fröhlich interaction as the carrier–substrate separation increases. Although the typical van der Waals gap is approximately 0.3–0.4 nm, experimental studies indicate that d can vary due to interface roughness, fabrication residues, or the intentional insertion of spacer layers, with effective values reaching up to ~1 nm [54]. Consequently, the range of d considered here is consistent with realistic device configurations and provides valuable insight into interface engineering strategies.

A comprehensive understanding of SOP interactions in monolayer PtS_2_ and PtSe_2_ requires careful consideration of the dielectric environment, particularly when these materials are supported on polar substrates such as SiO_2_ and hBN. The substrate’s dielectric response plays a dual role: it screens Coulomb interactions, thereby modulating excitonic binding energies and carrier mobility, while simultaneously introducing remote SOP modes that create additional scattering channels.

## 8. Discussion and Contextualization

Recent studies highlight the growing interest in substrate-dependent carrier dynamics in two-dimensional materials. In particular, theoretical investigations have examined electron–SOP interactions in monolayer TMDCs supported on dielectric substrates [17], as well as the interplay between Auger recombination and carrier–surface phonon interactions in graphene/TMDC heterostructures [18]. On the experimental side, PtSe_2_-based heterostructures have demonstrated promising optoelectronic performance, illustrating the technological relevance of these systems [11]. Furthermore, recent theoretical work has shown that the optoelectronic properties of PtS_2_/PtSe_2_ heterostructures can be tuned through strain engineering [12], while phonon-assisted Auger decay mechanisms in doped TMDC monolayers have also been investigated [17]. Together, these studies provide an important framework for understanding the carrier relaxation mechanisms analyzed in the present work.

Although the present study is theoretical, recent experimental investigations provide qualitative support for the mechanisms discussed here. In particular, experimental studies on PtSe_2_-based heterostructures have demonstrated that substrate properties can strongly influence carrier transport and optoelectronic response. For example, a graphene/PtSe_2_/ultrathin SiO_2_/Si broadband photodetector exhibiting high responsivity and fast response time has been reported [11]. These observations are consistent with our theoretical results, which show that the interaction between charge carriers in the monolayer and SOPs originating from the supporting substrate depends strongly on the dielectric properties of the latter. In addition, recent theoretical studies indicate that dielectric screening and strain engineering can significantly modify the electronic and optical properties of PtS_2_/PtSe_2_ systems [12], while phonon-assisted Auger decay processes in doped TMDC monolayers have been analyzed in Ref. [19].

These observations are consistent with a growing body of theoretical and experimental work highlighting the pivotal role of the dielectric environment in two-dimensional TMDCs. For instance, Gopalan et al. [55] examined how surrounding dielectrics—including substrates, gate insulators, and metallic electrodes—affect charge transport in monolayer TMDs. By combining ab initio calculations with a dielectric continuum model, they showed that while high-κ dielectrics can screen electron–phonon interactions and thereby enhance carrier mobility, this effect may be partially offset by additional scattering arising from remote phonon–plasmon modes. Their study also identifies hBN as a particularly favorable dielectric environment and systematically explores how material polarity, phonon resonances, carrier density, and temperature influence carrier transport properties.

Similarly, Hauber et al. [56] developed a self-consistent framework describing carrier scattering mediated by coupled phonon–plasmon excitations, incorporating both dynamic screening and anharmonic decay processes. Applied to MoS_2_-based heterostructures, their model demonstrates that dynamic screening can significantly enhance carrier mobility compared with static approximations, while the finite thickness of the dielectric interface can strongly influence transport behavior in polar two-dimensional environments.

Such substrate sensitivity is expected to be particularly pronounced in PtS_2_ and PtSe_2_ due to their relatively high polarizability and quasi-flat electronic bands, which enhance their susceptibility to perturbations originating from the supporting dielectric environment. Experimental observations support this general behavior. For instance, Kizel et al. [57] reported a polarization-dependent photoluminescence contrast of up to 400% in rhombohedral MoS_2_ multilayers induced by Fermi-level shifts in asymmetric dielectric environments. Although their work focused on ferroelectric-induced exciton–trion imbalance, the mechanisms discussed here for PtS_2_ and PtSe_2_—arising from coupling to SOPs in polar substrates such as SiO_2_ and hBN—reflect the same general principle that interfacial dielectric interactions can strongly modify optical responses in two-dimensional semiconductors.

Further studies emphasize the importance of interface-specific dielectric engineering in controlling carrier dynamics in TMDC-based heterostructures. Adeniran and Liu [58] demonstrated that dielectric screening at TMD/hBN interfaces differs significantly between monolayer and bulk regimes due to local-field effects, highlighting the importance of spatially resolved dielectric properties for both transport and optical behavior. Knobloch et al. [59] examined the limitations of ultrathin hBN as a gate dielectric, showing that while hBN provides an atomically smooth and low-defect interface suitable for materials such as PtS_2_ and PtSe_2_, extremely thin layers may lead to increased tunneling leakage. In addition, Wang et al. [60] further confirmed that substrate choice (e.g., SiO_2_ versus hBN) strongly affects carrier polarity and transport efficiency, underscoring the tunable nature of electron–phonon interactions via substrate engineering. Froehlicher et al. [61] demonstrated that interfacial interactions in graphene–TMDC heterostructures can strongly modify electronic and optical properties, suggesting by analogy that substrate effects may similarly influence electron–SOP coupling in PtS_2_ and PtSe_2_ monolayers on SiO_2_ and hBN.

The results obtained in the present work extend these previous studies by explicitly quantifying how SOP modes influence both the OS and the scattering dynamics in PtS_2_ and PtSe_2_ monolayers. In the calculations presented here, idealized interfaces between the monolayer and the dielectric substrate are considered in order to isolate the intrinsic mechanisms governing carrier–phonon interactions. In realistic experimental systems, additional factors such as defects, disorder, and interface roughness may influence the quantitative values of the scattering rates. Nevertheless, the qualitative trends predicted by the model—particularly the dependence of the relaxation mechanisms on the dielectric properties of the substrate and on carrier density—remain robust and provide useful insight for interpreting experimental observations.

The effects of substrate phonon energy, dielectric constant, and interfacial proximity are shown to play a decisive role in shaping polaronic behavior in supported monolayers. In particular, the formation of SOP-induced polaronic states governs energy relaxation processes in the two-dimensional layer. By controlling the strength of this interaction through substrate selection, it becomes possible to tune phonon-assisted hot-carrier dynamics. Such control represents an important design strategy for optimizing the performance of two-dimensional optoelectronic devices, including photodetectors, photovoltaic systems, and other high-speed nanoelectronic applications.

## 9. Auger Recombination

Auger recombination represents a fundamental non-radiative relaxation pathway in which the energy released from an electron–hole pair annihilation is transferred to a third carrier instead of being emitted as a photon [62,63,64,65]. This mechanism plays a central role in regulating photoluminescence efficiency and strongly affects the operation of light-emitting devices and photodetectors through its impact on energy transfer dynamics. Under specific conditions, Auger recombination may also enable carrier multiplication, thereby enhancing device responsivity. In two-dimensional semiconductors, and particularly in TMDCs, Auger scattering exerts a pronounced influence on the optical response by contributing both to energy dissipation and, in some cases, to additional carrier generation, with far-reaching implications for photonic and optoelectronic applications.

Although the present study is theoretical, Auger recombination processes in two-dimensional semiconductors are commonly investigated experimentally using time-resolved spectroscopic techniques such as ultrafast pump–probe spectroscopy and time-resolved photoluminescence measurements. These techniques enable the identification of non-radiative carrier relaxation channels and the estimation of Auger recombination rates. The theoretical predictions presented here regarding the dependence of Auger recombination on substrate dielectric properties and temperature could therefore provide useful guidance for future experimental studies of supported PtSe_2_ and PtS_2_ monolayers [11,12,19].

In monolayer TMDCs such as PtS_2_ and PtSe_2_, non-radiative Auger recombination constitutes a key channel for carrier relaxation. In these processes, the energy released by electron–hole recombination is transferred to a third carrier, leading to a redistribution of energy and momentum within the electronic system. When these monolayers are supported on polar substrates such as SiO_2_ or hBN, the dielectric screening of the surrounding environment modifies Coulomb interactions, thereby affecting both the strength and efficiency of Auger scattering.

**Figure 10:**
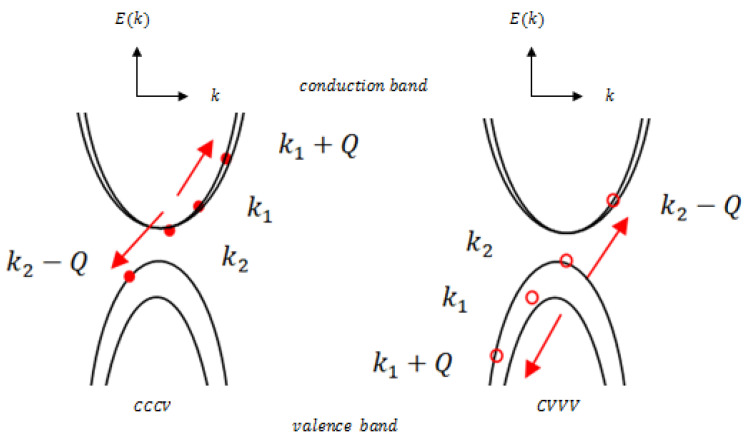
Auger scattering process in PtS_2_ and PtSe_2_ via CCCV and CVVV channels.

Electron–hole recombination mediated by Coulomb interactions in TMDCs proceeds through two distinct Auger channels, illustrated in Figure 10. In the CCCV process, an electron in the conduction band with momentum $k_{1}$ scatters with another conduction-band electron of momentum $k_{2}$. The final state consists of one electron in the conduction band with momentum $k_{1}+Q$ and another promoted to the valence band with momentum $k_{2}-Q$. Conversely, in the CVVV process, a hole in the valence band with initial momentum ***k***_1_ scatters with another valence-band hole of momentum $k_{2}$, resulting in one hole remaining in the valence band with momentum $k_{1}+Q$ and another promoted to the conduction band with momentum $k_{2}-Q$. These two processes are mirror-symmetric to each other and together govern the efficiency of Auger-driven carrier dynamics in PtS_2_ and PtSe_2_ monolayers.

To ensure a consistent treatment of the dielectric environment across the different scattering channels, we distinguish between the dynamic and static screening regimes relevant to SOP interactions and Auger recombination. SOP scattering originates from dynamical polarization fields in the polar substrate. Its coupling strength is determined by the contrast between the substrate’s high-frequency and static dielectric responses, which enters through the Fröhlich interaction as:

(12) $$\left(\frac{1}{\varepsilon_{\infty }+\varepsilon_{env}}-\frac{1}{\varepsilon_{0}+\varepsilon_{env}}\right)$$

Capturing the long-range evanescent fields generated by remote optical phonons. These fields dominate the small-(q) coupling experienced by carriers in supported two-dimensional semiconductors.

In contrast, Auger recombination is governed by statically screened Coulomb interactions, for which the relevant dielectric response is the low-frequency electrostatic screening of the monolayer–substrate heterostructure. We therefore use the standard effective dielectric constant $\bar{\varepsilon }=\frac{\left(\varepsilon_{sub}+\varepsilon_{env}\right)}{2}$, leading to the statically screened 2D Coulomb potential:

(13) $$V_{scr}\left(q\right)=\frac{e^{2}}{2\bar{\varepsilon }\;\left(q+q_{s}\right)}$$

where $q_{s}$ is the 2D Thomas–Fermi screening wavevector, which quantifies the ability of free carriers to screen long-range Coulomb interactions.

Although the mathematical expressions differ, they represent the same dielectric environment evaluated in the frequency regime intrinsic to each process—dynamic screening for SOP scattering and static screening for Auger recombination—ensuring a physically coherent and internally consistent description of environmental screening in all parts of the model.

For a 2D electron gas with parabolic band dispersion, the parameter $q_{s}$ is given by:

(14) $$q_{s}=\frac{e^{2}g_{s}g_{v}m^{*}}{\bar{\varepsilon }\hbar^{2}}$$

where $g_{s}$ and $g_{v}$ are the spin and valley degeneracies, respectively, and $m^{*}$ is the effective carrier mass. This expression emphasizes that, in two dimensions, the Thomas–Fermi wavevector is determined solely by the effective mass, degeneracy factors, and the dielectric environment, rather than by the carrier density. Consequently, $q_{s}$ provides a fundamental parameter for assessing the efficiency of electrostatic screening in supported 2D materials such as PtS_2_ and PtSe_2_.

The free-carrier Auger scattering rate in monolayer PtS_2_ and PtSe_2_ can be expressed using Fermi’s golden rule as [66]:

(15) $$\frac{1}{\tau_{Auger}}=\frac{2}{\hbar^{2}v}\int_{0}^{\infty }\frac{dk_{1}}{2\pi }\int_{0}^{\infty }\frac{dk_{2}}{2\pi }\int_{k_{2}}^{\infty }\frac{dQ}{2\pi }{\left|M\left(k_{1},k_{2},Q\right)\right|}^{2}\sqrt{\left(k_{1}+Q\right)\left(Q-k_{2}\right)k_{1}k_{2}}\;\left(1-f_{-1}\left(Q-k_{2}\right)\right)\left(1-\;f_{+1}\left(k_{1}+Q\right)\right)f_{+1}\left(k_{1}\right)f_{+1}\left(k_{2}\right)$$

where $\tau_{Auger}$ is the carrier lifetime associated with the Auger process, $f_{s}\left(k\right)$ are the Fermi–Dirac distribution functions, and v is the carrier group velocity along the relevant direction, defined from the band dispersion $v=\frac{\hbar k}{m^{*}}$.

This formulation ensures energy and momentum conservation, and the integrals can be numerically evaluated using the effective masses and dielectric environment of PtS_2_ and PtSe_2_.

The screened Coulomb interaction $M\left(k_{1},k_{2},Q\right)$ is expressed as the difference between direct and exchange contributions, and can be written as [64]:

(16) $$\left|M\left(k_{1},k_{2},Q\right)\right|=\;\left|M_{d}\left(k_{1},k_{2},Q\right)\right|-\left|M_{e}\left(k_{1},k_{2},Q\right)\right|$$

(17) $$M_{d}\left(k_{1},k_{2},Q\right)=V_{scr}\left(Q\right)F_{d}\left(k_{1},k_{2},Q\right)$$

(18) $$M_{e}\left(k_{1},k_{2},Q\right)=V_{scr}\left(Q+k_{1}-k_{2}\right)$$

In the present study, the evaluation of the screened Coulomb matrix elements near the K and K′ valleys requires the consideration of Bloch state overlap factors, denoted as $F_{d}$ and $F_{e}$. These factors quantify the degree of overlap between initial and final Bloch wave functions involved in the scattering process. In the long-wavelength limit and for intravalley processes, the overlap between Bloch states at nearby momenta is known to approach unity, with corrections that scale quadratically with the transferred momentum. This behavior can be rigorously understood within the framework of the quantum geometric tensor, where the expansion of the Bloch overlap reveals that deviations from unity are governed by the quantum metric and become negligible for small momentum transfers. Consequently, when evaluating Coulomb interactions close to the K and K′ valleys of monolayer PtS_2_ and PtSe_2_, it is well justified to approximate both $F_{d}$ and $F_{e}$ as unity in a first-order estimate. This approximation is consistent with recent theoretical insights that relate the real-space quantum metric of solids to the overlap of neighboring Bloch states [67]. Hence, the expression of the screened Coulomb interaction in terms of the 2D Thomas–Fermi wavevector $q_{s}$ remains accurate at the level of approximation adopted in this work.

Building upon this framework, we further assume that the occupation statistics of electrons in the conduction and valence bands follow the Fermi–Dirac distribution functions, expressed as

(19) $$f_{s}\left(k\right)=\frac{1}{1+e^{\left(E_{s}\left(k\right)-E_{f}\right)/k_{B}Τ}}$$

where s=+1 and s=−1 label the conduction and valence bands, respectively. $E_{s}\left(k\right)$ denotes the energy dispersion in band, $E_{f}$ is the Fermi energy, $k_{B}$ the Boltzmann constant, and T the temperature. This statistical description provides the basis for evaluating carrier populations and their contribution to Coulomb-mediated scattering processes.

The band dispersions near the K and K′ valleys in PtS_2_ and PtSe_2_ are approximated as parabolic:

(20) $$E_{c}\left(k\right)=\frac{∆}{2}+\frac{\hbar^{2}k^{2}}{2m_{c}^{*}}$$

(21) $$E_{v}\left(k\right)=-\frac{∆}{2}+\frac{\hbar^{2}k^{2}}{2m_{v}^{*}}$$

with ∆ as the direct gap at K and K′, and $m_{c}^{*}$ and $m_{v}^{*}$ as the effective masses for conduction and valence bands, respectively.

**Figure 11:**
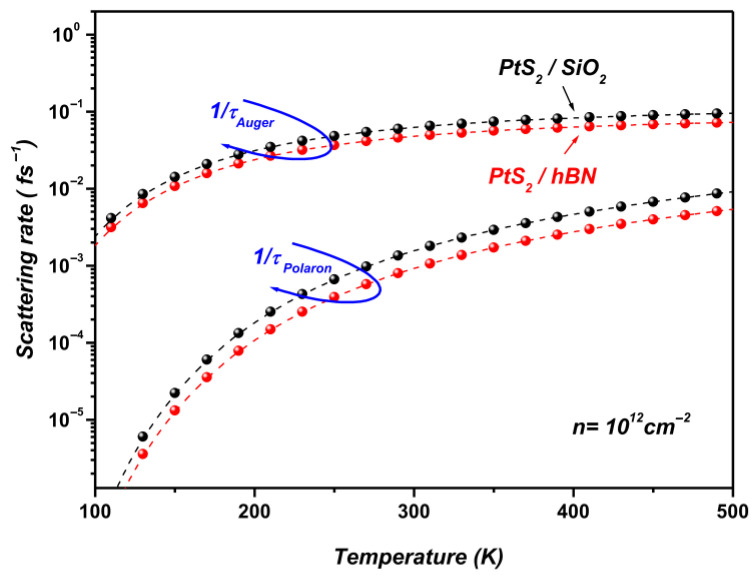
The temperature dependence of the Auger and SOP scattering rates in PtS_2_/SiO_2_ and PtS_2_/hBN interfaces, with a charge carrier density of $n=10^{12}\;{cm}^{-2}$.

Figure 11 presents the temperature dependence of the Auger and SOP scattering rates in PtS_2_/SiO_2_ and PtS_2_/hBN heterostructures, evaluated at a carrier density of $n=10^{12}\;{cm}^{-2}$. The carrier density used in the present calculations (${n=10}^{12}\;{cm}^{-2}$) corresponds to a representative value commonly employed in theoretical and experimental studies of two-dimensional semiconductor systems. Carrier densities in the range $10^{11}\;{cm}^{-2}$–$10^{13}\;{cm}^{-2}$ are typically achieved in field-effect devices and optical experiments involving TMDC monolayers. In general, the Auger recombination rate increases with carrier density because Auger processes involve interactions between multiple carriers. In many two-dimensional semiconductor systems, the Auger recombination rate increases with carrier density since the process involves multi-carrier interactions. As a result, the probability of carrier–carrier scattering events becomes larger, leading to an enhancement of the Auger recombination rate. Similar behavior has been reported in previous theoretical investigations of Auger recombination and carrier–surface phonon interactions in two-dimensional systems [18,29]. In contrast, the SOP scattering rate depends mainly on the electron–phonon coupling strength and the phonon population, and therefore exhibits a weaker dependence on carrier density.

The results demonstrate that both Auger and SOP scattering processes become increasingly pronounced at room temperature and above, whereas their contributions remain negligible at lower temperatures.

The results show that both Auger recombination and SOP scattering increase with temperature for the considered PtS_2_/SiO_2_ and PtS_2_/hBN heterostructures. Within the investigated temperature range (100–500 K) and for the carrier density used in the calculations ($n=10^{12}\;{cm}^{-2}$), the Auger scattering rate remains larger than the polaron-related scattering rate, indicating that many-body recombination processes play an important role in carrier relaxation under the present conditions. Nevertheless, the pronounced temperature dependence observed for both mechanisms indicates that carrier relaxation in these supported monolayers is governed by the combined influence of phonon-mediated and carrier–carrier interaction processes. These results highlight the role of temperature and dielectric environment in determining the dominant relaxation pathways in supported Pt-based TMDC monolayers.

Beyond their temperature dependence, the calculated scattering rates also provide insight into the transport behavior of carriers in supported monolayers. Since the carrier relaxation time is inversely proportional to the scattering rate, an increase in SOP or Auger-mediated scattering leads to shorter relaxation times and therefore reduced carrier mobility. Such substrate-induced transport limitations are well known in supported two-dimensional systems, where remote polar phonons originating from polar substrates can become a dominant scattering mechanism at elevated temperatures [23,24]. Similar conclusions have also been reported for phonon-limited transport in transition-metal dichalcogenide monolayers [25,26].

At higher temperatures, the calculated scattering rates tend to saturate as illustrated in Figure 11. For the PtS_2_/hBN interface, the Auger and SOP scattering rates converge to $\sim 0.07\;fs^{-1}$ and $\sim 0.005\;fs^{-1}$, corresponding to carrier lifetimes of approximately ~14.29 fs and ~200 fs, respectively. Similar convergence behavior has been reported in previous studies [18,27,28,29,52] on graphene supported by polar substrates under both low- and high-bias conditions. These works show that low-field mobility becomes nearly temperature-independent at high temperatures due to current saturation. In this transport regime, elastic scattering dominates at low fields, while current saturation arises from inelastic processes involving either substrate-induced surface polar phonons (SPPs) or the intrinsic optical phonons of graphene. Furthermore, high-bias experiments in graphene, as noted in [52], demonstrated that the magnitude of the saturated current is determined by the energy of the optical phonons driving the saturation. By analogy, the convergence of scattering rates observed here for PtS_2_ on polar substrates can likewise be attributed to such inelastic mechanisms.

In the present study, this effect is evident in the convergence of both Auger and SOP scattering rates at the PtS_2_/hBN and PtS_2_/SiO_2_ interfaces (Figure 11). Similar to graphene-based heterostructures, current saturation in PtS_2_ supported on polar substrates is primarily governed by inelastic scattering with SOPs and intrinsic optical phonons of PtS_2_. Additionally, electron overheating at elevated electronic temperatures contributes to this saturation, leading the system toward a steady-state regime in which further increases in the applied electric field no longer enhance the current.

In addition to their impact on carrier transport, the optical response of PtS_2_/hBN and PtS_2_/SiO_2_ heterostructures is strongly governed by Auger recombination and SOP interactions. At high carrier densities or in defect-rich environments, Auger processes suppress photoluminescence, which may limit the efficiency of light-emitting devices but can enhance carrier multiplication in photodetectors. A key aspect revealed in this study is the saturation of both Auger and SOP scattering rates at elevated temperatures, a behavior analogous to that observed in graphene on polar substrates. This convergence arises from inelastic scattering processes involving surface polar phonons of the substrate or intrinsic optical phonons of the 2D layer, driving the system into a steady-state regime where further increases in temperature or electric field no longer significantly affect carrier dynamics. In hybrid heterostructures such as PtS_2_/hBN, PtS_2_/SiO_2_, PtSe_2_/hBN, and PtSe_2_/SiO_2_, interlayer coupling and external fields provide additional means to control Auger dynamics, phonon-mediated scattering, and saturation effects. In this context, both polaronic effects and Auger-mediated carrier relaxation emerge as central mechanisms for optimizing high-performance two-dimensional optoelectronic devices, including photodetectors and solar cells.

The systematic comparison between PtSe_2_ and PtS_2_ monolayers supported on SiO_2_ and hBN demonstrates that the dielectric environment plays a crucial role in determining the strength of the interfacial electron–phonon interaction. In particular, the larger anticrossing gaps obtained for hBN-supported structures indicate stronger effective coupling between electronic states in the monolayer and the polar phonon modes of the substrate. This behavior reflects the sensitivity of the Fröhlich-type interaction to the dielectric properties and phonon spectra of the supporting substrate.

Moreover, the comparative analysis between SOP scattering and Auger recombination reveals a crossover between phonon-mediated and many-body carrier relaxation mechanisms that depends on temperature and carrier density. These results provide insight into how the dielectric environment and intrinsic material properties jointly govern carrier relaxation dynamics in supported Pt-based TMDC monolayers.

The results obtained in the present study further highlight the important role of dielectric engineering in controlling carrier relaxation processes in supported two-dimensional semiconductors. In particular, the stronger anticrossing behavior and the larger Rabi splitting observed for hBN-supported structures indicate that the dielectric environment can significantly modify the strength of the interfacial electron–phonon interaction. Such substrate-dependent effects are consistent with previous theoretical and experimental studies showing that polar substrates can strongly influence carrier mobility, scattering rates, and hot-carrier relaxation in two-dimensional materials through remote phonon coupling mechanisms [23,24,25]. From a device perspective, these findings suggest that the choice of substrate can serve as an effective strategy to tune carrier relaxation pathways and optimize the performance of PtSe_2_- and PtS_2_-based optoelectronic and photodetection devices.

Taken together, these results provide a coherent physical picture of how substrate-induced SOPs and Auger recombination jointly influence carrier relaxation and transport processes in supported PtSe_2_ and PtS_2_ monolayers.

## 10. Conclusions

In summary, we have presented a comprehensive theoretical investigation of electron–SOP interactions and Auger recombination processes in monolayer PtSe_2_ and PtS_2_ supported on polar dielectric substrates. Our analysis demonstrates that substrate-induced SOPs play a central role in governing carrier relaxation dynamics in these supported two-dimensional systems. The hybridization between electronic excitations and SOPs leads to the formation of polaronic states characterized by pronounced anticrossing behavior and significant Rabi splitting. The magnitude of these effects strongly depends on the dielectric properties of the supporting substrate. In particular, the larger anticrossing gaps predicted for hBN-supported structures indicate stronger effective electron–phonon coupling compared with SiO_2_-supported systems.

Furthermore, the comparison between SOP scattering and Auger recombination reveals that carrier relaxation in these materials arises from the interplay between phonon-mediated scattering and many-body recombination processes. Within the investigated temperature range, Auger recombination constitutes the dominant relaxation channel, whereas phonon-mediated scattering becomes increasingly significant at elevated temperatures, where both mechanisms approach a comparable inelastic phonon-limited regime. These results highlight the fundamental role of the dielectric environment in shaping carrier dynamics in supported TMDC monolayers and emphasize the potential of substrate engineering as an effective strategy for controlling carrier relaxation pathways and transport properties.

More broadly, the present results provide physically grounded predictions on how the dielectric environment influences electron–phonon coupling and carrier relaxation mechanisms in Pt-based TMDC heterostructures. Several quantities predicted in this work—such as substrate-dependent anticrossing gaps, the temperature dependence of the scattering rates, and the competition between phonon-mediated and Auger relaxation processes—are experimentally accessible through optical spectroscopy and transport measurements. The theoretical framework developed in this study therefore provides useful guidance for future experimental investigations and for the design and optimization of next-generation TMDC-based optoelectronic and photonic devices.

Overall, the present work contributes to a deeper understanding of carrier relaxation mechanisms in supported two-dimensional semiconductors and highlights the importance of dielectric engineering for controlling electron–phonon interactions and many-body effects in emerging TMDC-based heterostructures.

## Figures and Tables

**Table 1:** Parameters used to model SOP scattering in monolayer PtS_2_ and PtSe_2_ on polar substrates.

	${SiO}_{2}$ ^*a*^	hBN ^*b*^
$\varepsilon_{0}$	3.9	5.09
$\varepsilon_{\infty }$	2.4	4.1
$\hbar \omega_{SO}\;\left(\mathrm{meV}\right)$	58.9	101.7
$\hbar \omega_{LO}\;\left(\mathrm{meV}\right)$	62.5	103.7
$F_{\nu }^{2}\;\left(\mathrm{meV}\right)$	0.237	0.258
$d\;\left(\mathrm{nm}\right)$	0.4	0.34

*^a^* References [23,40]. *^b^* References [23,48].

## Data Availability

The original contributions presented in this study are included in the article. Further inquiries can be directed to the corresponding author.
